# Chronic deep brain stimulation of the medial forebrain bundle reverses depressive-like behavior in a hemiparkinsonian rodent model

**DOI:** 10.1007/s00221-015-4375-9

**Published:** 2015-07-21

**Authors:** Luciano L. Furlanetti, Volker A. Coenen, Iñigo A. Aranda, Máté D. Döbrössy

**Affiliations:** Laboratory of Stereotaxy and Interventional Neurosciences, Department of Stereotactic and Functional Neurosurgery, University Freiburg-Medical Center, Breisacher Str. 64, 79106 Freiburg, Germany

**Keywords:** Chronic mild stress, Deep brain stimulation, Depression, Medial forebrain bundle, Rat model

## Abstract

Preclinical and clinical evidence suggests that depression might be associated with a dysfunction in the reward/motivation circuitry. Deep brain stimulation (DBS) of the superolateral branch of the medial forebrain bundle (MFB) has been shown in a recent clinical trial to provide a prompt and consistent improvement of depressive symptoms in treatment-resistant patients. In order to better understand the underlying mechanisms of neuromodulation in the context of depression, the effects of chronic bilateral MFB-DBS were assessed in a combined rodent model of depression and Parkinson’s disease. Female Sprague-Dawley rats received unilateral 6-OHDA injection in the right MFB and were divided into three groups: CMS-STIM, CMS-noSTIM and control group. The CMS groups were submitted to chronic unpredictable mild stress (CMS) protocol for 6 weeks. MFB-DBS was applied only to the CMS-STIM group for 1 week. All groups were repeatedly probed on a series of behavioral tasks following each intervention, and to a postmortem histological analysis. CMS led to an increase in immobility in the forced swim test, to a decrease in sucrose solution consumption in the sucrose preference test, as well as to an increased production of ultrasonic vocalizations in the 22 kHz range, indicating increased negative affect. MFB-DBS reversed the anhedonic-like and despair-like behaviors. The results suggest that unilateral dopamine depletion did not preclude MFB-DBS in reversing depressive-like and anhedonic-like behavior in the rodent. Further understanding of the importance of hemispheric dominance in neuropsychiatric disorders is essential in order to optimize stimulation as a therapeutic strategy in these diseases.

## Introduction

Neuropsychiatric manifestations of Parkinson’s disease (PD) have been associated to as much, if not more, morbidity as its classic motor features. A high prevalence of major depression in this population has been reported (Wolters and Baumann 2013). In spite of a range of medical and nonmedical therapies available for the treatment of neuropsychiatric disorders, some of these current options are insufficient and lead patients to suboptimal outcome (Holtzheimer et al. 2012; Aarsland et al. 2014). During the last decade, neuromodulation has emerged as a potential therapeutic option and several methods that have invasive and noninvasive techniques have been described (Krack et al. 2010; Holtzheimer et al. 2012). Neuromodulation via deep brain stimulation (DBS) has shown in recent trials proof of principal in reversing symptoms of drug-resistant neuropsychiatric disorders, such as obsessive–compulsive disorder (OCD), Tourette syndrome, addiction and major depression (MDD) (Krack et al. 2010). Although many hypotheses have been proposed concerning the mechanisms and neurocircuitries involved, the lack of a consensus on the underlying pathology is reflected in the variety of DBS targets proposed in different studies. Clinical trials and experimental works have most commonly investigated the nucleus accumbens (NAC), subcallosal cingulate gyrus (SCG) and ventral capsule/ventral striatum (VC/VS) as options. However, if on the one hand the indications in favor to a specific target overlap across the different disorders and, on the other hand, the average success rate for any of them for DBS does not overcome 50–60 %, doubts emerge as to the correctness of the targeted structures. Recently, an open-label clinical trial in (nonparkinsonian) chronically depressed patients stimulated the superolateral branch of the medial forebrain bundle (sIMFB), a structure that projects and interacts with all the previously selected targets. Stimulation provided a prompt and long-lasting improvement of depressive symptoms (Schlaepfer et al. 2013). Previous preclinical research of our group has shown that high-frequency electric stimulation (HFS) of the MFB leads to an increased expression of the early gene *c*-*fos* in all the same brain areas previously aimed as a single target, showing a simultaneous and immediate effect of MFB stimulation on several distant key points of the mesolimbic and mesocortical system (Furlanetti et al. 2015b; Döbrössy et al. 2015). These findings have been reproduced by other authors also showing MFB-DBS to widely increase neuronal activity in cortical and subcortical structures involved in the underlying circuitry of depression (Bregman et al. 2015), in contrast to activation limited to cortical or subcortical areas when targeting, for instance, the medial prefrontal cortex or the NAC (Hamani et al. 2014). This reinforces the rationale for MFB-DBS in the treatment of MDD and confirms that, rather than stimulation of a single target, it might be understood as neuromodulation of multiple afferent and efferent connections, inducing short- and long-term adaptations in local and distal neuronal activity in key areas implicated in the neurocircuitry of depression.

In order to better understand the pathology of depression and in an attempt to optimize medical treatment, animal models of depression have been applied in preclinical research. One way of establishing these models is by inducing key symptoms of the disease that are manifested as depressive-like behavioral phenotype. For example, the continuous exposure to a sequence of mild stressors, such as small periods of food deprivation and cage tilting, leads rats to develop depressive-like behavior that can be evaluated according to the animal’s performance in specific behavior testings (Willner 2005). In our previous clinical research, we have speculated that slMFB DBS in MDD is effective because of a bilateral activation of the ventral tegmental area (VTA) via recruitment of glutamatergic descending fibers of the MFB that in turn might change DA-metabolism in the remote nodes of the network (Schlaepfer et al. 2013, 2014). The aim of the current experiment was to first evaluate the effects of chronic and continuous bilateral MFB-DBS on a validated rodent model of depression, and secondly to investigate if unilateral depletion of dopamine in the nigrostriatal, mesocortico and mesolimbic pathways would preclude MFB neuromodulation of rescuing rat’s normal phenotype.

Our results validate the CMS model and provide data, showing that stimulation of the MFB was effective in reversing despair- and anhedonic-like behavior, despite almost complete unilateral absence of dopaminergic neurons.

## Materials and methods

### Study design

**Fig. 1:**
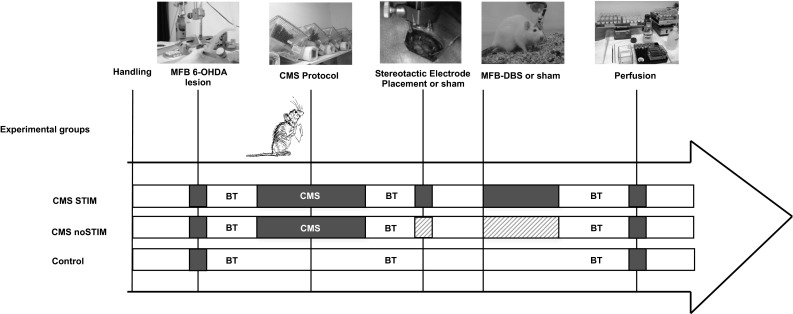
Experimental groups and study design. *MFB* medial forebrain bundle, *6-OHDA* 6-hydroxydopamine, *CMS* chronic mild stress, *DBS* deep brain stimulation, *BT* behavior tests. *Gray patterned areas* indicate “sham” procedure

Young adult female Sprague-Dawley (SD) rats (*n* = 13, Charles River, Germany), weighing 250 g, were housed in individual round cages (height: 40 cm; diameter: 40 cm), with the light/dark cycle maintained at 12 h on and 12 h off with food and water available *ad libitum*. After 2 weeks of handling and habituation, rats (*n* = 13) received a unilateral lesion via stereotactic injection of 6-hydroxydopamine (6-OHDA) in the right medial forebrain bundle (MFB), leading to complete dopamine depletion in the ipsilateral nigrostriatal and mesolimbic pathways. Following 18 months, baseline behavior tests were performed. Subjects that did not reach the inclusion criteria of more than 5 net ipsilateral rotation/min after amphetamine administration and <30 % use of the contralateral paw on the cylinder test were excluded from the study. Based on their performance, groups were matched and animals (*n* = 9) selected for chronic mild stress protocol or to compose the control group. After six consecutive weeks of CMS, groups were once again matched and animals fell into three groups, i.e., CMS-STIM (*n* = 5), CMS-noSTIM (*n* = 4) or control (*n* = 4). Before the final round of behavior tests, 1 week of continuous MFB-DBS (CMS-STIM group) or sham stimulation (CMS-noSTIM group) was performed. Then, animals were finally transcardially perfused, and brains prepared for histological assessment. The study described in this manuscript was approved by the local veterinary authorities (*Regierungspraesidium*; TVA G10-124) and was carried out in accordance with the EU Directive 2010/63/EU concerning the protection of animals used for scientific purposes. Experimental groups and design are summarized in Fig. 1.

### Chronic mild stress protocol

The animals (CMS-STIM, CMS-noSTIM groups) were exposed to a variety of mild stresses, such as disruption of light/dark cycle, cage tilting, isolation and paired housing, stroboscopic light, soiled cage, brief food/ water restriction and white noise, for a maximum of 6 consecutive weeks. Monitoring of health status and animal’s welfare by weight measurements (twice a week) and sucrose preference test (SPT) in a weekly basis looking at changes in hedonic experiencing were addressed during this period (Willner et al. 1987; Forbes et al. 1996).

### Surgical procedures

Rats underwent general anesthesia induced and maintained by inhalation of isoflurane 2 %. First, 6-OHDA (3.6 µg/µl in 0.2 % ascorbic acid and 0.9 % saline; Sigma, USA) was unilaterally injected in two tracks in the MFB at the following coordinates relative to bregma: track 1, tooth bar (TB) set at = +3.4; anteroposterior (AP) = −4.0; lateral (L) = −0.8; dorsoventral (DV) = −8.0; and track 2, TB = −2.3; AP = −4.4; L = −0.8; DV = −7.8. In total, each animal received 5.5 µl of solution at a rate of 1 µl/min. In a second time point, bipolar electrodes (125 µm diameter each, 90 % platinum/10 % iridium, 15-mm-length Teflon-coated shaft, World Precision Instruments, Sarasota, USA) were stereotactically bilaterally implanted into the MFB and permanently fixed to the skull surface with microscrews and bone cement. Anterior–posterior (AP) and mediolateral (ML) coordinates are taken from bregma, and DV coordinate from dura. AP = −4.4, ML = ±1.2, DV = −7.8. A single-shot buprenorphine (75 μg/Kg, i.p.) was given to all animals for postoperative analgesia.

### Deep brain stimulation

Following 2 weeks of postsurgical recovery, the groups were matched and animals connected to the pulse generator (STG 2008, Multichannel Systems, Germany) for stimulation (square-wave biphasic constant current, 130 Hz, 100 μs and 250 µA average amperage) or sham stimulation, as previously described elsewhere (Furlanetti et al. 2015a). The current was individually titrated for each cerebral hemisphere, beginning with 50 μA to a maximum of 350 μA. If unwanted behaviors were observed during the titration process, the current was set 50 µA less than the level that provoked side effects (e.g., rotational behavior, dystonic movements). Chronic and continuous MFB-DBS was performed for 7 consecutive days. The CMS-noSTIM group had burr holes drilled on their heads, but electrodes were not implanted. A third group served as control for health status monitoring and behavior tests.

### Behavioral assessment

Behavioral tests were performed at three different time points along the experiment, i.e., (1) baseline (following unilateral 6-OHDA injection); (2) post-CMS protocol; and (3) post-MFB-DBS.

#### Ultrasonic vocalization test (USV)

Rats emit distinct types of USVs, depending on age, environmental factors and the subject’s current emotional state. Low-frequency vocalizations (around 22 kHz) reflect a negative affective or aversive behavior, whilst ultrasonic vocalizations (USVs) in the high-frequency range (50 kHz) correlate with pleasurable or rewarding experiences (Knutson et al. 1998; Burgdorf et al. 2000; Burgdorf and Panksepp 2006; Wöhr et al. 2008; Sadananda et al. 2008). Testing was performed in individual Plexiglas cages with the recording microphones, sensitive to frequencies of 15–100 kHz placed 60 cm above the bottom and connected to an UltrasoundGate (Metris, Netherlands) and finally to a computer. Usual environmental conditions were maintained during the 2 h of continuous recording. Behavior analysis was performed using an automated evaluation system (Sonotrack 2.0, Metris, Netherlands). The number of events that occurred in each frequency range during the experiment was acquired for statistical assessment.

#### Forced swim test (FST)

Forced swim test (FST) is typically used to assess the antidepressant effect of drugs or other treatments in rodents (Porsolt et al. 2001; Overstreet 2012; Slattery and Cryan 2012). The protocol consists of placing the animal into a cylindrical receptacle (40 cm high, 20 cm diameter) filled with water (25 °C) and measuring the time of activity and immobility. The water level was adjusted so that the rat could not touch the bottom of the container with its tail and could not escape from the cylinder either. Behavior activity was recorded for 7 min by a digital video camera connected to the workstation (Viewer^2^, Biobserve, Germany). The amount of time spent in a posture of immobility was calculated. In accordance with the literature, immobility was defined as: (1) no movement of the three out four paws, (2) no struggling, (3) floating behavior.

#### Sucrose preference test (SPT)

The SPT assess anhedonia-like behavior or better the hedonic response in rodents. It was performed at the same time points as the other tests, plus in a weekly basis during the CMS protocol. Reduced preference for the sucrose solution indicates a decreased sensitivity to rewarding experiences and is understood as a preclinical correlate of anhedonia (Willner et al. 1987; Willner 2005). During this test, rats were offered for 24 h 2 bottles containing 400 ml, one with 5 % sucrose solution and the other tap water. Prior to the test, animals were not deprived of food or water. The amount of consumed water and sucrose solution was then calculated and presented as a percentage of the total volume.

#### Drug-induced rotation

 Rats were placed in rotameter bowls connected to a computer. A customized system was adapted from the original design of Ungerstedt (1971), allowing the test to be carried out under continuous DBS. Ipsilateral and contralateral rotations were recorded over 90 min after amphetamine injection (2.5 mg/kg i.p., Sigma; in 0.9 % saline). Data are presented as net ipsilateral or contralateral turns/min.

#### Cylinder test

Forelimb movement asymmetry and exploratory behavior were tested with the cylinder test (Schallert et al. 2000; Cordeiro et al. 2014) following MFB lesion (BL) and in the other sessions indicated above. The animals were placed in the center of a 20-cm-diameter clear Plexiglas cylinder, and their exploratory behavior was recorded with a camera for 4 min. The number of times the animal made clear contact with the cylinder wall, using either the ipsilateral or the contralateral forepaw, was counted, and the data expressed as the percentage of contralateral paw use with respect to the total touches made.

### Immunohistochemistry and histological analysis

Following the final round of behavior tests, animals were terminally anesthetized by an overdose of 10 % ketamine (Bela-Pharm GmbH & Co. KG, Germany) and 2 % xylazine (Rompun, Bayer-Leverkusen, Germany) and intracardially perfused with a solution containing 4 % paraformaldehyde and 0.025 % glutaraldehyde in 0.1 M phosphate buffer at pH 7.4. The brains were removed from the skull, kept in 30 % sucrose at 4 °C until they sunk, and cut into 40 μm coronal sections. The free-floating sections were incubated with 1.5 % H_2_O_2_, 1 % sodium borohydride and 1 % milk powder, each in 0.02 M sodium phosphate buffer at pH 7.4 for 30 min, and exposed to a primary antibody raised in goat against *c*-*fos* (SC-52-G, 1:2000, Santa Cruz Biotechnology Inc., Santa Cruz, USA), or mouse anti-tyrosine hydroxylase (TH) (1:2500; Sigma-Aldrich, Steinheim, Germany), or rabbit anti-serotonin (1:2000; Sigma S5545, Darmstadt, Germany). After incubation for 48 h at 4 °C, visualization of antibody-binding sites was based on DAB staining using biotinylated anti-goat (BA-5000; 1:200; Vector Laboratories, Inc., Burlingame, USA) or biotinylated anti-mouse (BA-2001; 1:200; Vector Laboratories, Inc., Burlingame, USA) as secondary antibody and avidin-biotin technique (ABC Elite; Vector Laboratories, Burlingame, CA) for signal intensification. Finally 3,3′-diaminobenzidine (DAB; Merk, Darmstadt, Germany) and 0.01 % H_2_O_2_ were used to develop the color reaction. The sections were mounted on super frost plus slides (Langenbrinck, Emmendingen, Germany), dehydrated in ascending alcohol solutions and cleared in xylene before they were coverslipped with Histofluid (Marienfeld Laborglas, Lauda-Königshofen, Germany).

Assessment of the final electrode position was carried out by overlapping the TH histological sections and the respective slice found on a standard stereotaxic rat brain atlas. *C*-*fos* (in the prelimbic frontal cortex, lateral habenular nucleus and shell of the accumbens) and 5-HT (in the dorsal raphe nucleus (DRN), prelimbic frontal cortex and accumbens) expressions were quantitatively assessed, using a Leica DMRB microscope and the Stereoinvestigator software (MFB Bioscience, USA). Immunoreactivity was estimated using Abercrombie’s correction formula *P* = *M*/(*D* + *M*)*A* × *N*, where *P* = total immunoreactivity, *M* = section thickness (here, 40 µm), *D* = average diameter of the stained structures, *A* = number of counted units and *N* = number of cut series (6).

### Statistics

Two- or three-way ANOVAs with repeated measures were used (Statistica, StatSoft, Inc., USA). In all cases, the main effects were tested for groups (CMS-STIM, CMS-noSTIM, control), and sessions (baseline, post-CMS, post-MFB-DBS). When appropriate, post hoc analyses were performed using Student–Newman–Keuls test. Level of significance was set at *p* < 0.05. Results are expressed as mean ± standard error of the mean (SEM).

## Results

**Fig. 2:**
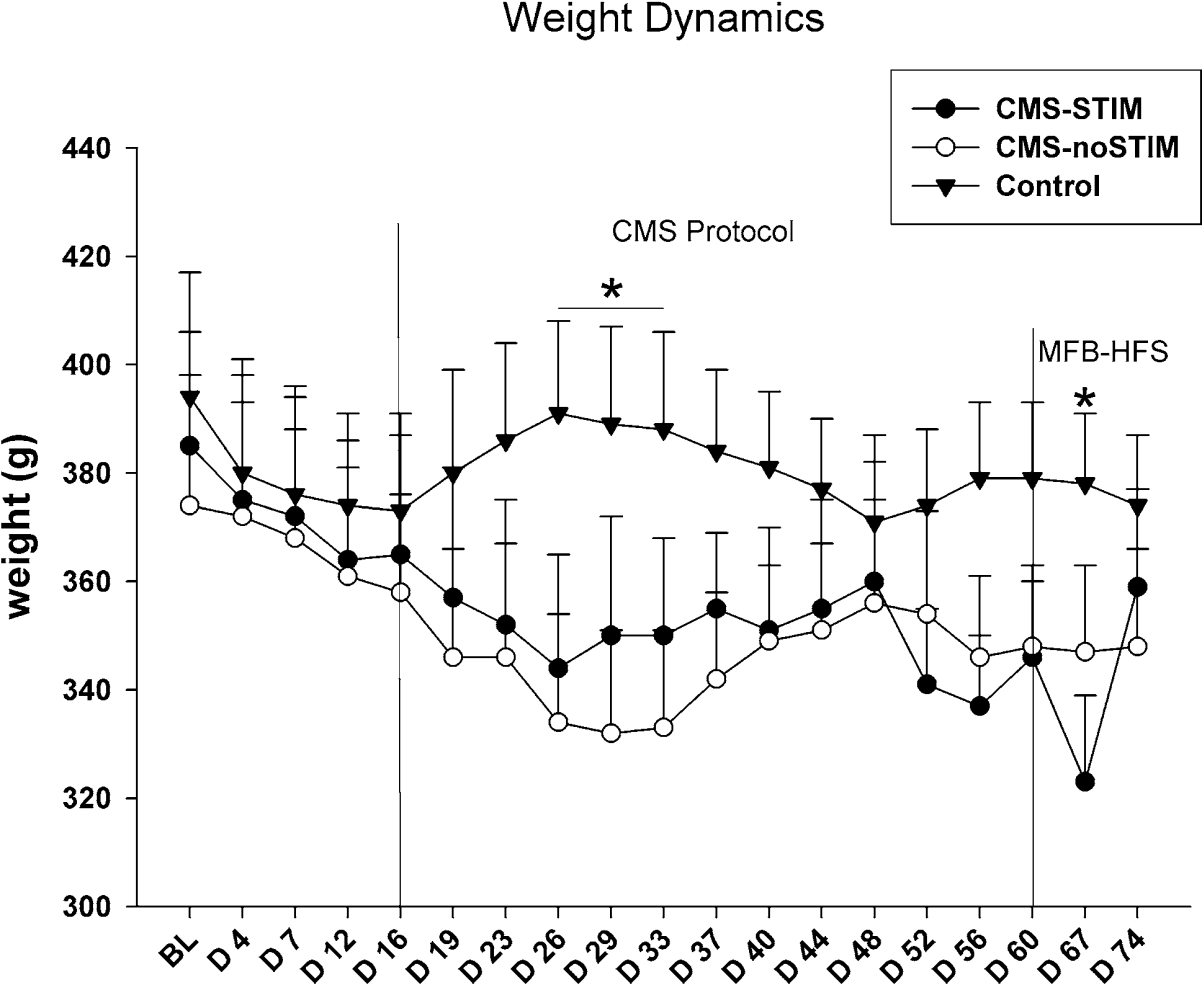
Weight dynamics. During the CMS protocol, statistical significance toward a mild loss of body weight in the CMS groups in comparison with the controls was observed (*p* < 0.01). MFB-DBS also led to a temporary decrease in weight in the CMS-STIM group; however a prompt recovery even during ongoing stimulation occurred (*p* = 0.01). *Asterisks* indicate statistical significance across groups

The weight of the animals was regularly monitored during the study. The CMS protocol did not have a significant bearing on the animals’ weight dynamics, but a mild and temporary reduction was observed (5–10 % of the initial body mass), similar to following the first days of MFB stimulation [group × session, *F* (18,180) = 2.44, *p* = 0.001]. After a variable period, the animals continued to gain weight again normally (Fig. 2).

### Behavioral assessment

**Fig. 3:**
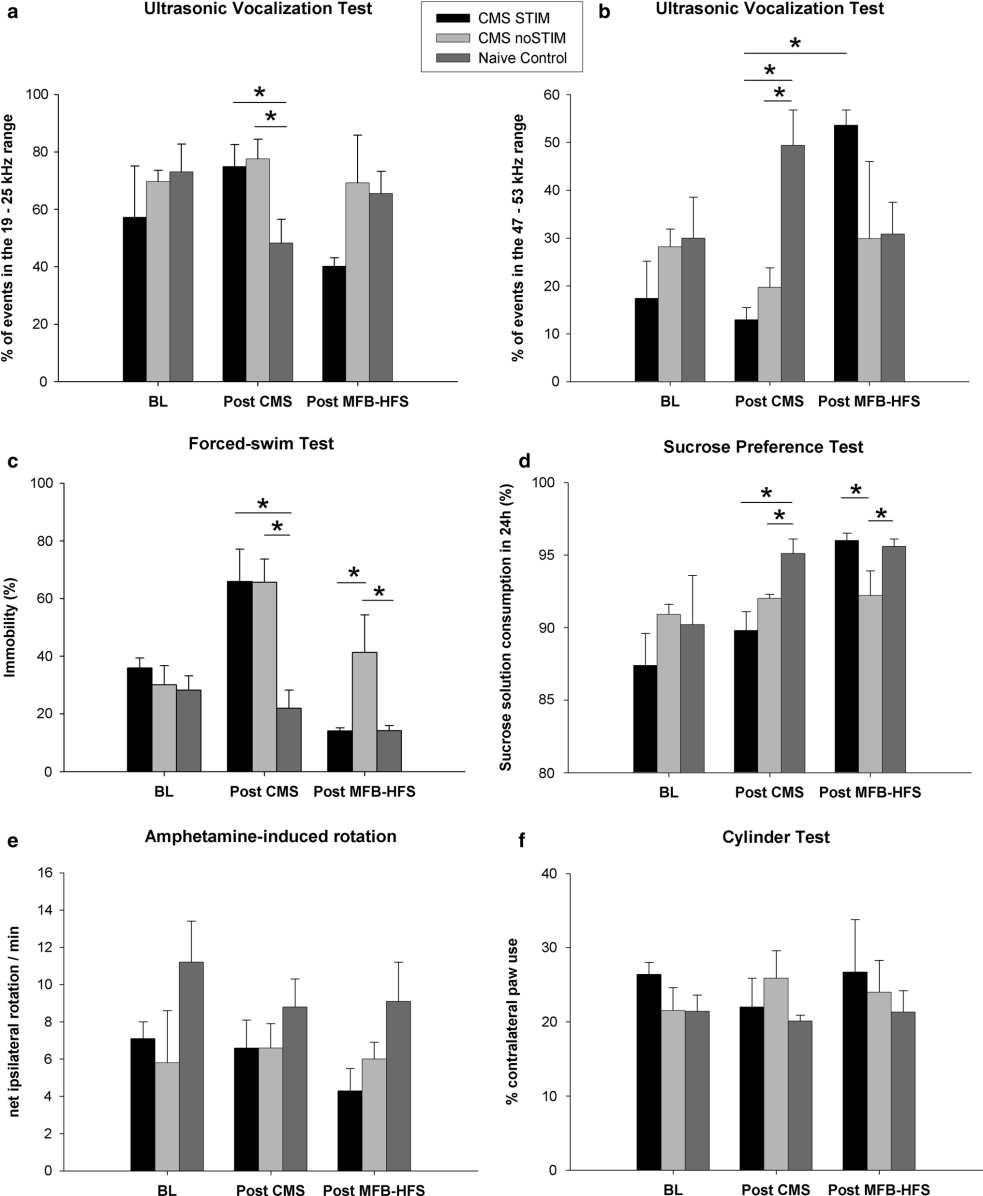
Behavioral testing. **a**, **b** Ultrasonic vocalization (USV): the chronic mild stress protocol (CMS) led to an increase in the number of calls at 22 kHz (CMS-STIM vs control, *p* = 0.03, CMS-noSTIM vs control, *p* = 0.05) as well as to a decrement of high-frequency calls (CMS-STIM vs control, *p* = 0.001, CMS-noSTIM, *p* = 0.001). Following MFB-DBS, a session effect was detected, with an increase of 50 kHz calls in the CMS-STIM group (*p* = 0.005); **c** forced swim test (FST): Exposure to mild stressors caused exacerbation of despair (CMS-STIM vs control, *p* = 0.0004, CMS-noSTIM vs control, *p* = 0.0003) that was reverted by chronic stimulation of the MFB to a level comparable with the control group (CMS-STIM vs control, *p* = 0.3, CMS-noSTIM vs control, *p* = 0.02, CMS-STIM vs CMS-noSTIM *p* = 0.003); **d** sucrose preference test (SPT): anhedonic-like behavior was induced by CMS (CMS-STIM vs control, *p* = 0.01, CMS-noSTIM vs control, *p* = 0.06, CMS-STIM vs CMS-noSTIM, *p* = 0.1) and rescued by MFB-DBS (CMS-STIM vs control, *p* = 0.8, CMS-noSTIM vs control, *p* = 0.02, CMS-STIM vs CMS-noSTIM, *p* = 0.03); **e**, **f** amphetamine-induced rotation and cylinder test: assessment of forelimb akinesia and drug-induced rotational behavior did not reveal any impact of neither the mild stressors nor the neuromodulation of the MFB on behavioral outcome (*p* = 0.6 and *p* = 0.1, respectively). *BL* baseline test, *post-CMS* behavior tests following the chronic mild stress protocol, *post-MFB-HFS* behavior assessment after chronic continuous high-frequency deep brain stimulation of the medial forebrain bundle. *Asterisks* indicate statistical significance across groups/sessions

Both motor and nonmotor behavioral aspects were assessed along the experiment. A baseline test session revealed in all groups a typical lateralized forelimb movement asymmetry due to unilateral dopamine depletion in the nigrostriatal and mesolimbic pathways, tested with amphetamine-induced rotation and cylinder tests. Neither improvement nor worsening of the motor deficits was observed following CMS protocol or MFB-DBS [Fig. 3e (amph-induced rotation), group × session, *F* (2,10) = 0.52, *p* = 0.6; Fig. 3f (cylinder test), group × session, *F* (2,10) = 2.87, *p* = 0.1].

Anhedonic behavior was measured using the SPT. The data show that all tested animals presented a similar percentage of sucrose solution consumption prior to CMS. Along the CMS protocol, both CMS-STIM and CMS-noSTIM groups showed a weekly decrement in sucrose preference with an overall 23 % drop by the end of CMS [Fig. 3d, group × session, *F* (4,20) = 2.98, *p* = 0.04]. This could be translated as installation of anhedonic-like behavior induced by a sequence of mild stressors. Chronic continuous stimulation of the MFB led, on the other hand, to a significant increase in the amount of sucrose solution drunk, showing strong positive effect on rescuing a phenotype comparable to the controls (*p* = 0.02). Similarly, performance on the FST, which evaluates behavioral despair, dropped 83 % in the groups submitted to the CMS protocol [Fig. 3c, group × session, *F* (4,20) = 9.18, *p* < 0.001], whilst 1 week of chronic and continuous MFB-DBS reduced immobility from 65.9 ± 11.2 to 6.1 ± 1.9 % (*p* = 0.0001).

The impact of CMS and DBS of the mesolimbic circuitry on USV, specifically on appetitive 50 kHz and aversive 22 kHz USVs, was also tested. Following the period of CMS, a significant increase in the number of aversive calls [Fig. 3a, group × session, *F* (2,10) = 4.34, *p* = 0.04], accompanied by a drop of events in the 50-kHz range, was observed (*p* = 0.04). Interestingly, once again MFB-DBS showed a strong effect toward an inversion of this profile, with 86 % reduction of 22 kHz calls, paralleled with an increase of 50 kHz USVs among the CMS-STIM [Fig. 3b, group × session, *F* (4,20) = 5.01, *p* = 0.005], while their nontreated counterparts (CMS-noSTIM) did not present any improvement (*p* = 0.5). These findings reveal a persistent aversive phenotype induced by chronic stress that was reversed by DBS.

### Histology

**Fig. 4:**
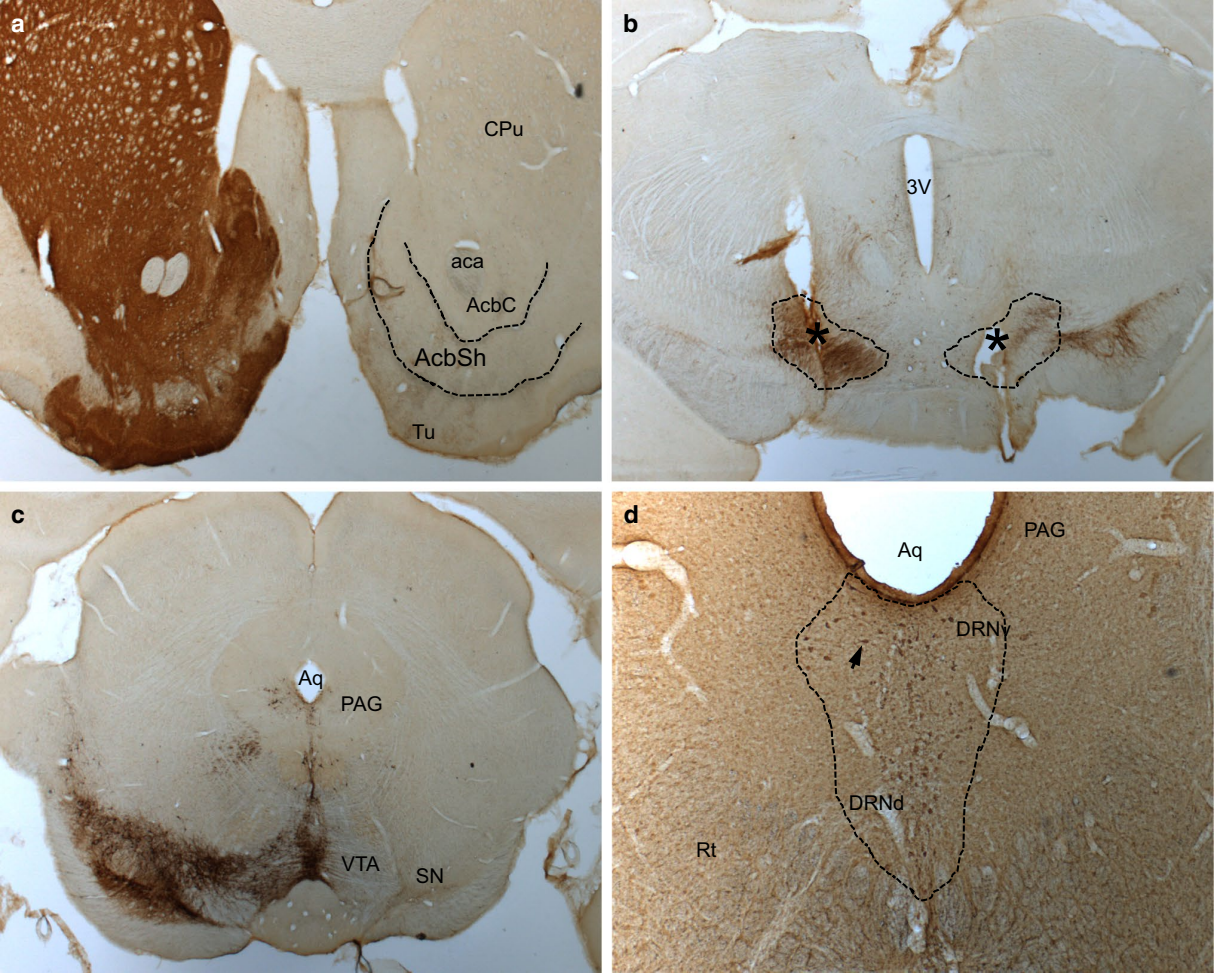
Tyrosine hydroxylase (TH) and serotonin (5HT) immunostainings. A complete unilateral dopamine depletion following the injection of 6-OHDA in the right medial forebrain bundle (MFB) is represented on micrographs (**a**–**c**). The asterisks indicate the tip of the stimulating electrodes placed bilaterally into the MBF (**b**). MFB-DBS led to an increase in the number of 5HT immunoreactivity in the dorsal raphe nucleus (DRN) (**d**). *Arrows* indicate positive staining in the DRN. *CPu* caudate-putamen (striatum), *aca* anterior commissure, *AcbSH* nucleus accumbens, shell, *AcbC* nucleus accumbens core, *3V* third ventricle, *Aq* aqueduct, *Tu* olfactory tubercle, *PAG* periaqueductal gray, *VTA* ventral tegmental area, *SN* substantia nigra, *DRNv* dorsal raphe nucleus, ventral part, *DRNd* dorsal raphe nucleus, dorsal part, *Rf* reticular formation

**Fig. 5:**
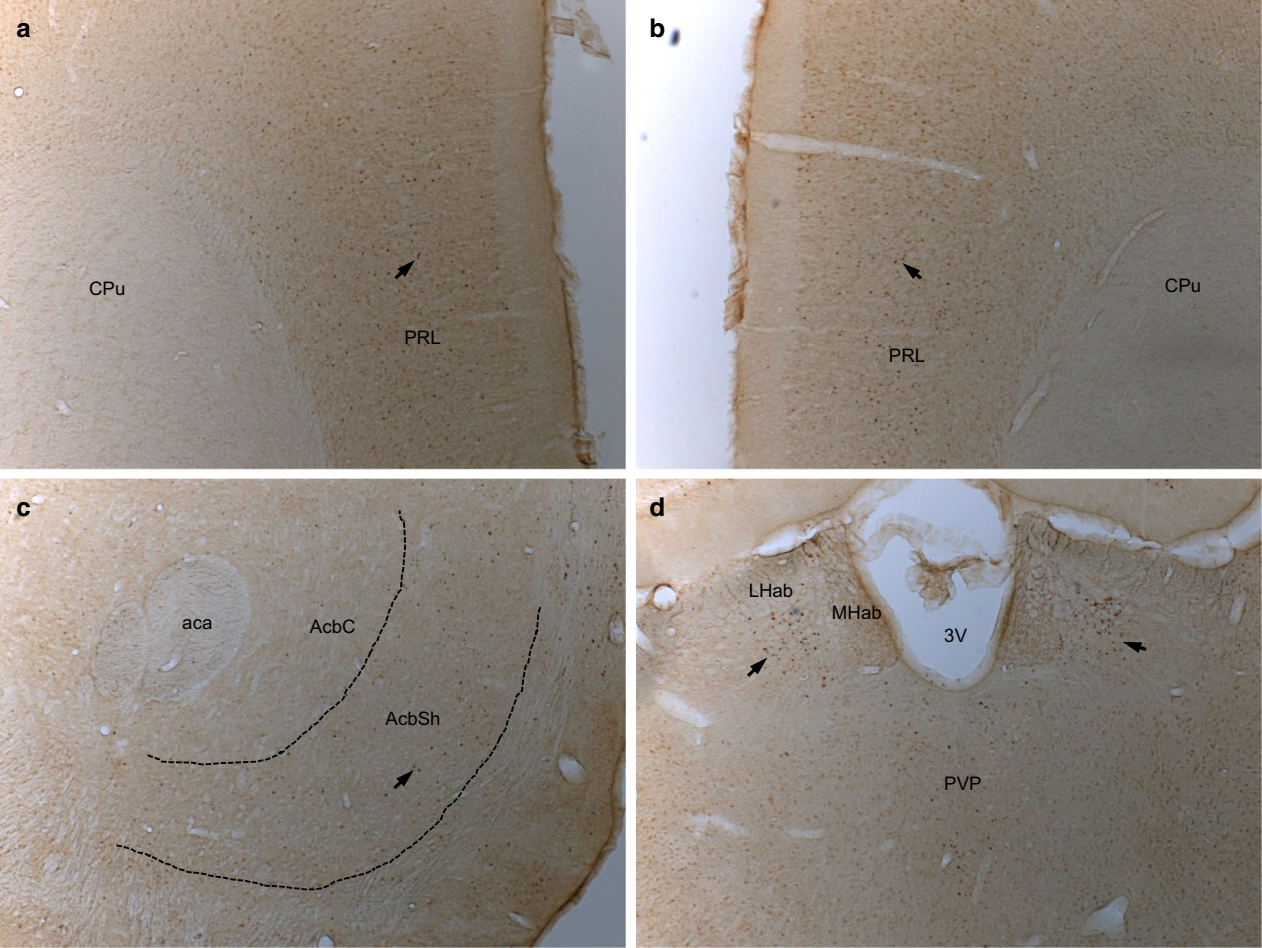
*c*-*fos* immunostaining following bilateral chronic continuous MFB-DBS. The micrographs show a strong expression of the early gene *c*-*fos* in the prelimbic frontal cortex (**a**–**b**), and mild upregulation of this marker in the shell of the nucleus accumbens and lateral habenular nucleus (**c**, **d**, respectively). *Arrows* indicate positive staining. *CPu* caudate-putamen (striatum), *PRL* prelimbic frontal cortex, *aca* anterior commissure, *AcbSH* nucleus accumbens, shell, *AcbC* nucleus accumbens Core, *3V* third ventricle; *LHab* lateral habenular nucleus, *MHab* medial habenular nucleus, *PVP* paraventricular thalamic nucleus

**Fig. 6:**
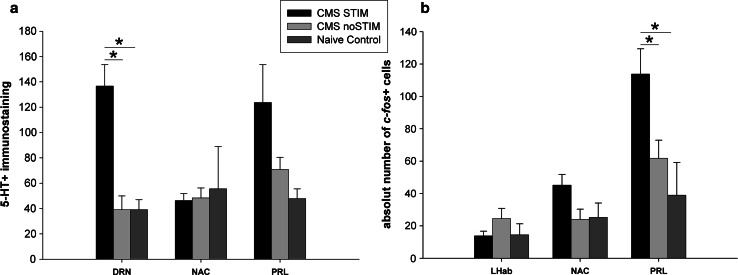
Stereological assessment of 5HT and *c*-*fos* expression following MFB-DBS. A significant increment of 5HT immunoreactivity in the DRN in the CMS-STIM group was observed (*p* < 0.001) (**a**). Although not statistically significant, MFB-DBS also led to a strong increase in 5HT immunolabeling in the prelimbic frontal cortex (PRL) (*p* = 0.07). Nevertheless, expression of the early gene *c*-*fos* in this same brain area was clearly upregulated (*p* = 0.01) and in the shell of the nucleus accumbens (NAC) also increased (*p* = 0.08) (**b**). No significant changes in *c*-*fos* activation in the lateral habenular nucleus or 5HT expression in the NAC were observed (*p* = 0.3 and *p* = 0.9, respectively)

The consistency and accuracy of the bilateral electrode implantation were addressed and confirmed on the TH staining, showing the tip of the electrodes within the borders of the MFB at the level of the lateral hypothalamus (LH) (Fig. 4b). A severe depletion of TH-positive neurons and fibers on the right nigrostriatal, mesolimbic and mesocortical pathways was observed (Fig. 4a–c). Interestingly, stereological analysis showed a significant increase in *c*-*fos* expression in the prelimbic frontal cortex (PRL) induced by selective stimulation of the MFB (Figs. 5a, b, 6b, *p* = 0.01). However, unilateral depletion of dopaminergic fibers via injection of 6-OHDA in the right MFB reduced the effect of MFB-DBS on activating *c*-*fos* expression in the shell of the NAC (Figs. 5c, 6b, *p* = 0.08). Compared to controls, MFB neuromodulation also induced a statistically significant 3.5-fold increase in serotoninergic immunoreactivity in the DRN (Fig. 6a, *p* < 0.001) and a strong, but not significant, increase in the PRL (*p* = 0.07).

## Discussion

Electrical stimulation of the superolateral branch of the medial forebrain bundle has been shown to produce rapid and long-lasting anti-depressive effects in major depression patients resistant to the conventional therapies (Schlaepfer et al. 2013; Coenen et al. 2013). Acute electrical stimulation targeting the MFB and associated pathways have been investigated in preclinical models of psychiatric disorders (Corbett and Wise 1980; Carlezon and Chartoff 2007; Sagara et al. 2010; Rea et al. 2014; Döbrössy et al. 2015); however, there is no general consensual hypothesis concerning the mechanistic explanations for how neuromodulation of the MFB impacts on the network model of depression (Mayberg 1997; Döbrössy et al. 2015) although groups have speculated about this (Schlaepfer et al. 2014). In the current experiment, a combined hemiparkinsonian and chronic mild stress model of depression was used to evaluate the behavioral impact of bilateral chronic continuous high-frequency stimulation of the MFB. Histological methods were employed to shed light on possible biological mechanisms. The results confirm that clinically relevant stimulation parameters of MFB-DBS induce recovery of anhedonic- and depressive-like behaviors in an established animal model of depression. Unilateral depletion of DA neurons did not preclude overall (and especially ipsilateral) neural activation and upregulation of c-fos expression in ascending target areas such as in the shell of the NAC and prelimbic frontal cortex (PRL), whilst MFB stimulation led to an increased expression of 5-HT both in the PRL and in the DRN, indicating that neurotransmitters other than dopamine might be involved, additionally.

### The chronic mild stress model of depression

Preclinical and experimental research using animal models of human diseases can shed light on the mechanisms of the diseases or help evaluate new treatment strategies. The etiology of depression is currently not understood but is considered to arise from the dysregulation of neuronal activity in numerous loci on the limbic-cortical circuit (Mayberg 1997). Two core symptoms of depression in humans are anhedonia and reduced motivation, which in the animal models of depression are assessed by the sucrose preference (SPT) and the FST, respectively. Since its introduction in the late 1980s, and the development of theories that stress and traumatic life events can promote the manifestation of depression (Kessler 1997; de Kloet et al. 2005), the CMS model has shown to provide a unique combination of predictive, face and construct validities and therefore has been used in numerous preclinical studies (Forbes et al. 1996; Willner 2005; Hamani and Temel 2012; Döbrössy et al. 2015). In the present experiment, continuous exposure of the animals to CMS procedures led to a weekly reduction in the preference for sucrose solution in the SPT, as well as to a significant increase in immobility in the FST, in comparison with the baseline tests and control animals (Willner et al. 1987; Papp et al. 1991; Muscat et al. 1992; Sikiric et al. 2000; Bielajew et al. 2003). Along with classic behavioral tests, UVS paradigms have also been applied in preclinical research for assessment of animal’s affective state (Knutson et al. 1998; Burgdorf et al. 2000; Wöhr and Schwarting 2007; Portfors 2007). Studies have indicated that, in rats, 22 kHz calls are closely associated with aversive events and, for instance, can be elicited by fear conditioning (Wöhr et al. 2005), handling the animal for the first time (Brudzynski and Ociepa 1992) or following social isolation (Francis 1977). Conversely, high-frequency USVs usually translate a positive affective state (Wöhr et al. 2008). Rats emit 50 kHz calls during mating (while approaching and investigating the partner) and also in a nonsexual context, in order to (re-)establish or to maintain contact among conspecifics (Burgdorf et al. 2000; Wöhr and Schwarting 2007). In line with this, in the present experiment, rats chronically exposed to stressors showed a decreased number of calls in the 50-kHz range and a higher number of aversive USV compared to the controls. Activation of the SEEKING system via neuromodulation of the MFB (Panksepp 1998; Coenen et al. 2011) likely led to 4.1-fold increase in the number of 50 kHz calls. This behavioral change was accompanied by increased serotonergic immunoreactivity in the DRN, as well as by a higher expression of the early gene *c*-*fos* in the PRL. Although the mechanisms underlying this observation are not completely clear, electrical stimulation of the ventral medial prefrontal cortex—that sends projections to the DRN via the MFB—has been shown to rescue animals from anti-depressive phenotype and induce increase in the size and density of serotonergic synapses (Veerakumar et al. 2014).

### MFB-DBS and the neurocircuitry of depression

The broad spectrum of different emotional, cognitive and autonomic symptoms observed in MDD patients appears to result from a dysfunction of the network rather than a defect of one specific region or imbalance of an particular pathway (Mayberg 1997; Panksepp 1998; Friedman et al. 2009; Coenen et al. 2012; Anderson et al. 2012; Döbrössy et al. 2015). It is generally thought that the ventral striatal area is the major subcortical site where affective processes are integrated with action and motor behaviors. The mesolimbic/mesocortical ascending DA pathways—connecting the VTA—to the NAC and the medial frontal cortex are considered as the neural substrate for the SEEKING system. It has been postulated that this system subserves reward-related, explorative behavior, including motivation and appetitive learning, and its dysfunction plays a key role in psychiatric disorders such as addiction, schizophrenia and depression (Panksepp 1998; Arnsten and Rubia 2012; Russo and Nestler 2013). Hyper-reactivity of this system in more complex settings could be present in addiction or certain forms of schizophrenia, whilst depression, anhedonia and lack of motivation are believed to be related to a hypoactivity of this pathway. Although over the last decade neuromodulation of numerous brain regions associated with the neurocircuitry of depression has been attempted, the reported remission rate does not overcome 30–50 % (Jiménez et al. 2005; Lozano et al. 2008; Malone et al. 2009; Sartorius et al. 2010; Kennedy et al. 2011; Puigdemont et al. 2012; Bewernick et al. 2012). On the other hand, bilateral stimulation of the superolateral branch of the MFB showed antidepressant efficacy in patients within days and with relatively low intensities of stimulation (Schlaepfer et al. 2013; Coenen et al. 2013). The reason for the near-immediate effect is not yet known; however, likely, it is related to the pivotal role of the MFB in modulating/synchronizing the function of all the other key structures implicated in the network (Döbrössy et al. 2015). In the present study, MFB-DBS led to an increased expression of the early gene *c*-*fos* in the shell of the NAC, as well as of 5-HT in the dorsal part of the raphe nucleus, and of both markers in the PRL. The data suggest a role for both the activation of distant structures in this circuit and also the implication of neurotransmitters other than dopamine in the antidepressant mechanisms of DBS in the treatment of depression (Hamani et al. 2010b, 2014). Therefore, neuromodulation of this system is considered as a potential therapeutic strategy in the treatment of resistant MDD (Coenen et al. 2011; Schlaepfer et al. 2013; Döbrössy et al. 2015).

### Potential mechanisms of action

Although experimental modeling of human mental illnesses in animals is crucial to better understand the neurobiological mechanisms of diseases and their treatment options, one should be aware of its limitations (Matthews et al. 2005). The present study, aiming to investigate the role of midbrain dopaminergic projections in bilateral MFB-DBS-mediated phenotypical rescue in a model of depression, used a unilateral lesion approach to disrupt dopamine availability in the NAC. Ideally, in order to address this issue, animals with bilateral 6-OHDA MFB lesions should have received bilateral MFB-DBS. However, it has been previously demonstrated that adult rats survive poorly following complete bilateral MFB lesions due to aphagia, adipsia and severe motor impairments (Deumens et al. 2002). Differential effects of lateralized stimulation have been investigated by others, suggesting that targeting the left, but not the right, medial prefrontal cortex is associated with antidepressant-like effect (Hamani et al. 2010a). On the other hand, lesion of the right, but not left, medial PFC was shown to lead to an anxiolytic effect with reduction in stress-induced corticosterone response via neuroendocrine and autonomic modulation (Sullivan and Gratton 1999, 2002).

Concrete evidence concerning the short- and long-term biological consequences of DBS in animal models is scarce. Noninvasive, in vivo proton magnetic resonance spectroscopy imaging has shown to elevate both glutamate and GABA levels in the striatum in unilateral dopamine-depleted hemiparkinsonian rats, and ipsilateral DBS of the subthalamic nucleus modified this state and normalized transmitter levels (Melon et al. 2015). Similarly, in the current paper, we speculate that the MFB stimulation could have both direct and indirect impacts on the activity of serotonergic and dopaminergic fibers.

Firstly, serotoninergic projections connecting the nucleus raphes dorsalis to the forebrain and to a number of areas of interest in the context of depression, such as mamillary body, lateral habenular nucleus, septum, hippocampal and preoptic regions, NAC and amygdaloid complex, travel through the MFB (Nieuwenhuys et al. 1982). MFB-DBS could modulate directly the activity of ascending serotonergic fibers in the MFB and increase transmission in the NAC and other target areas. In our experiments, we have observed that MFB-DBS has led to an upstream and downstream modulation of those fibers with consequently overexpression of 5HT in key areas of the limbic network, namely the DRN and the medial PFC.

However, MFB-DBS could also have secondary effect on dopaminergic transmission in the NAC: Recruitment of myelinated glutamatergic fibers that descend from the PFC and the striatum toward the VTA could increase tonic DA output (on the intact side) from the VTA to its ascending targets and lead to the reduction in the depressive phenotype. The upregulation of the early gene *c*-*fos* in the shell of the NAC and medial PFC seen in the study would confirm the increased activity in these distal targets following the stimulation.

In summary, although the actual mode of action of MFB-DBS remains unclear, evidences allow us to speculate toward the combined neuromodulation of multiple monoaminergic systems implicated in the network of depression. Whether unilateral modulation of the MFB with DBS would be sufficient for the management of TRD in humans remains to be investigated.

## Conclusion

The benefits of DBS in the management of treatment-resistant neuropsychiatric disorders have been presented over the last decade and remain under evaluation both in terms of effectiveness and safety. Not only the mechanisms of neuromodulation as an alternative treatment, but also the underlying neuropathology of the disease are under investigation and still need to be clarified. Neuromodulation in animal models of depression has been proven to be an important tool for addressing these issues. Recent clinical work targeting the MFB by DBS in treatment-resistant depression confirmed this bundle as a promising target that deserves attention in preclinical studies. The current experiment demonstrated that unilateral dopamine depletion does not preclude MFB-DBS in reversing depressive-like and anhedonic-like behavior. Upregulation of c-fos expression and increased 5-HT in key regions of the circuitry have been associated with behavioral improvement. Taken together, MFB-DBS should not be understood as an intervention on a simple target, but as neuromodulation of a whole circuitry, since neurotransmitters other than dopamine are additionally involved. This confirms the strategic position of the MFB as its stimulation possibly recruits multiple afferent and efferent connections, inducing short- and long-term plastic adaptations in local and distal neuronal activity.
